# Vagus nerve size determined via ultrasonography is associated with white matter lesions in patients with vascular risk factors

**DOI:** 10.1007/s40477-024-00936-2

**Published:** 2024-07-29

**Authors:** Tomohisa Nezu, Futoshi Eto, Akemi Hironaka, Shiro Aoki, Shuichiro Neshige, Saki Tasaka, Hikari Kirimoto, Hirofumi Maruyama

**Affiliations:** 1 Department of Clinical Neuroscience and Therapeutics, Hiroshima University Graduate School of Biomedical and Health Sciences, 1-2-3 Kasumi, Minami-Ku, Hiroshima, 734-8551 Japan; 2 Department of Sensorimotor Neuroscience, Hiroshima University Graduate School of Biomedical and Health Sciences, Hiroshima, Japan

**Keywords:** Vagus nerve size, White matter lesions, Ultrasonography, Hypertension

## Abstract

**Purpose:**

The cross-sectional area (CSA) of the cervical vagus nerve (VN), as assessed through ultrasonography, might be linked to autonomic nervous system dysfunction. Hypertension is the primary factor associated with cerebral white matter lesions (WMLs), but there is also evidence of a connection with autonomic nervous system dysfunction. However, the associations between WMLs and VN size are unclear. Our objective was to investigate the associations between WMLs and VN size in patients with vascular risk factors.

**Methods:**

The CSA of the VN was evaluated using carotid ultrasonography in patients with a history of stroke (acute or chronic) and comorbidities (*n* = 196, 70.2 ± 12.7 years). Common carotid artery (CCA) intima-media thickness and interadventitial diameter (IAD) were also measured. The severity of the WMLs was assessed by the Fazekas classification and Scheltens’ scale.

**Results:**

The CSA of the right VN (2.08 ± 0.65 mm^2^) was significantly greater than that of the CSA of the left VN (1.56 ± 0.44 mm^2^) (*P* < 0.001). Multiple linear regression analyses revealed that older age, hypertension, increased right CCA IAD, and decreased CSA of the right VN (standardized partial regression coefficient [β] − 0.226; *P* < 0.001) were independently associated with the severity of WMLs (Scheltens’ scale). A decreased CSA of the left VN was also associated with the severity of WMLs (β = − 0.239; *P* < 0.001).

**Conclusion:**

VN size determined via ultrasonography was associated with the severity of WMLs. While these findings do not establish a causal relationship, they suggest that autonomic nervous system dysfunction is involved in the progression of WMLs.

**Supplementary Information:**

The online version contains supplementary material available at 10.1007/s40477-024-00936-2.

## Introduction

The cervical vagus nerve (CVN) is typically located dorsomedially between the common or internal carotid artery and the internal jugular vein. The main component of CVN fibers is the parasympathetic nervous system, which modulates heart rate, respiratory rate, and gastrointestinal activity. CVN afferent fibers influence the nucleus tractus solitarius and the dorsal motor nucleus of the vagus nerve. Subsequently, these nuclei directly or indirectly project to various brain regions, including the locus coeruleus, dorsal raphe nucleus, amygdala, hippocampus, and cerebral cortex [1]. The VN plays various roles in transmitting bidirectional signals between the brain and peripheral organs via autonomic function, anti-inflammatory mechanisms, and regulation of blood–brain barrier permeability [2]. VN stimulation has been applied in the treatment of various central nervous system diseases, such as epilepsy, depression, anxiety, and migraine, and in the rehabilitation of stroke patients [2].

Recently, high-frequency ultrasonography has been utilized to evaluate CVN morphology noninvasively. Several studies have been conducted to verify the normal values of the cross-sectional area (CSA) of the CVN in healthy individuals [3]. Several cross-sectional studies found that a decreased CSA of the CVN was associated with a diagnosis or the progression of Parkinson’s disease [4, 5]. In addition, a decreased CSA of the CVN was also observed in patients with diabetes mellitus [6].

The common pathophysiology in both diabetes mellitus and Parkinson's disease is autonomic nervous system dysfunction. These patients sometimes suffer from symptoms of blood pressure fluctuations, such as orthostatic hypotension [7]. Several studies have shown that blood pressure variability and orthostatic hypotension are associated with the progression of cerebral white matter lesions (WMLs) [8, 9]. WMLs, which are considered a cerebral small vessel disease, are known to cause cognitive impairment, dementia and disability [10, 11]. Blood pressure variability, including orthostatic hypotension, is associated with cognitive decline and a greater risk of dementia [12–14]. The rostral ventrolateral medulla (RVLM), which is involved in the regulation of the autonomic nervous system, is an important central site for the maintenance of sympathetic vasomotor tone and blood pressure regulation [15]. We previously reported that RVLM compression was associated with increased blood pressure variability among acute ischemic stroke patients [16]. In that study, RVLM compression was mildly associated with WMLs. To date, there have been no reports on whether autonomic dysfunction, as reflected by a decreased VN size or RVLM compression, is associated with the severity of WMLs. The aim of this study was to investigate the associations among VN size, RVLM compression and the severity of WMLs in patients with vascular risk factors.

## Methods

### Subjects

This was a single-center hospital-based retrospective study. Patients with cerebrovascular disease (acute stroke or chronic stroke) and other atypical neurological problems, such as dizziness, underwent a clinically indicated ultrasonographic examination of their carotid artery systems between February 2020 and December 2023 at Hiroshima University Hospital. Of the 756 patients who underwent carotid ultrasonography for the evaluation of carotid atherosclerosis, the CSA of the VN was evaluated for 209 patients in typical clinical settings. Of these 209 patients, 200 patients who underwent magnetic resonance imaging (MRI) in typical clinical settings within 6 months before or after the day when carotid ultrasonography was performed were included in this study. Patients with a history of Parkinson’s disease (*n* = 3) or inflammatory peripheral neuropathy (*n* = 1) were excluded. Finally, 196 patients were analyzed in the present study. Baseline clinical characteristics, including age, sex, height, body weight, body mass index (BMI), hypertension, diabetes mellitus, dyslipidemia, atrial fibrillation and history of stroke (ischemic or hemorrhagic), were recorded. The criteria for hypertension, diabetes mellitus, dyslipidemia, and atrial fibrillation were previously defined [17]. The study conformed to the Declaration of Helsinki and was approved by the ethics committees at Hiroshima University, including the institutional review board (approval number E2023-0201). This study was performed under the opt-out method, as it was performed using clinical records. Informed consent for participation was not obtained from the participants.

### Carotid ultrasonography

#### Vagus nerve size by ultrasonography

**Fig. 1:**
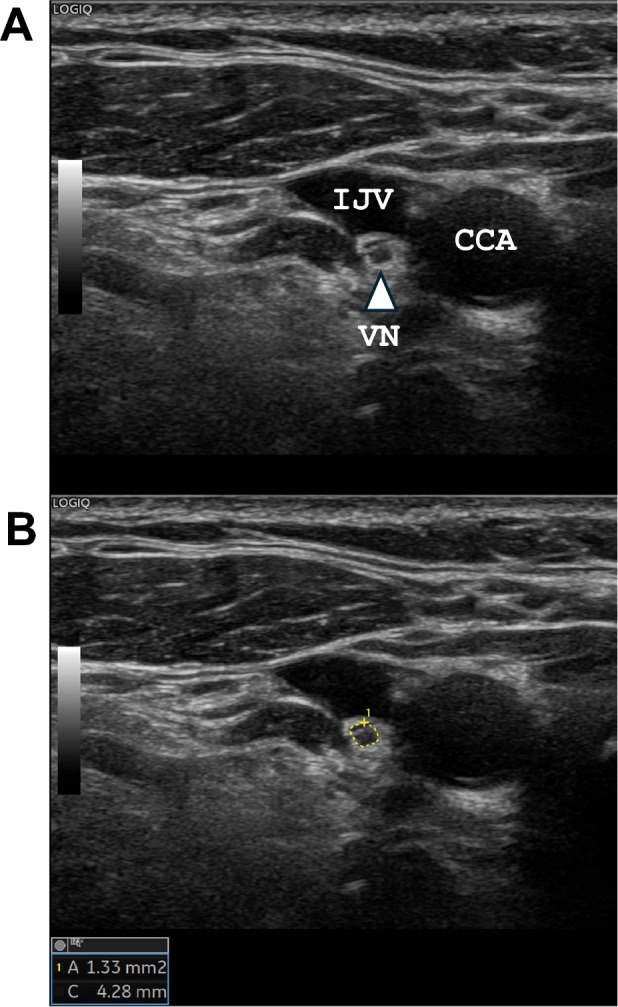
Ultrasonographic image of the vagus nerve (VN). The right VN is shown as a small, rounded, hypoechoic structure between the common carotid artery (CCA) and the internal jugular vein (IJV) (**A**, triangle arrow). The cross-sectional area of the VN was measured by manual tracing (**B**, yellow circle)

Ultrasound was performed to evaluate the CSA of the VN using a LOGIQTME9 XD clearTM2.0 (GE Healthcare, Wauwatosa, WI, USA) with a 10–15 MHz linear array transducer. Ultrasonography and measurements of the CSA of the VN were performed by one of three experienced technicians (T.N., F.E., and A.H.) who were certified by the Japan Academy of Neurosonology. Cross-sectional imaging of the VN was recorded bilaterally at the level of the thyroid gland. The VN was identified within the carotid sheath between the internal jugular vein and the carotid artery, with the transducer placed transversely on the lateral neck (Fig. 1A). During the study, all subjects were placed in a supine position and rotate their heads away from the scanning side. The CSA of the VN was obtained using the trace method, measuring along the inner aspect of the hyperechoic epineurium (Fig. 1B). The CSA of the VN (right VN and left VN) was measured twice by technicians, and the mean CSA was calculated. Between each measurement, echo transducer was completely removal. The intraclass correlation coefficient (ICC) (1, 2) was consistently high for each technician (T.N.: *n* = 125, 0.956 for right VN, 0.938 for left VN; F.E.: *n* = 43, 0.776 for right VN, 0.828 for left VN; A.H.: *n* = 28, 0.937 for right VN, 0.878 for left VN). For the interrater correlation, ICC (2.1) for the CSA of the VN was calculated for 10 patients in a preliminary study using a different cohort as follows: between T.N. and A.H., 0.878 for right VN and 0.823 for left VN; between T.N. and F.E., 0.922 for right VN and 0.928 for left VN.

#### Carotid artery measurements by ultrasonography

The common carotid arteries (CCAs) were evaluated using high-resolution B-mode duplex ultrasonography with a 7.5-MHz linear probe. Optimal bilateral visualization of the carotid artery was performed with the patients lying in the supine position and their necks slightly extended. We measured the maximal intima-media thickness (IMT) at the far wall of the distal CCA (CCA-IMT), which is the 10-mm section of the artery proximal to the starting point of the carotid bulb. In addition, we measured the common carotid interadventitial diameter (IAD) on both sides.

### Magnetic resonance imaging

MRI was performed with a 3.0 T scanner (SIGNA, GE Medical Systems, Fairfield, CT, USA or Philips Ingenia, Philips Medical Systems, Best, the Netherlands). The imaging protocol consisted of T1-weighted spin‒echo, T2-weighted spin‒echo and fluid-attenuated inversion recovery (FLAIR) sequences. The severity of the WMLs was rated visually on the FLAIR images using the Fazekas scale as follows: no lesions, grade 0; punctate lesions, grade 1; early confluent lesions, grade 2; and confluent lesions, grade 3 [18]. Periventricular hyperintensity (PVH) and deep white matter hyperintensity (DWMH) were graded separately on FLAIR or T2-weighted spin‒echo images according to the Fazekas scale. In addition, deep white matter changes (frontal, temporal, parietal, and occipital lobes) were assessed using Scheltens’ scale, with the total possible score ranging from 0 to 24. Periventricular white matter changes (range, 0–6) were assigned scores of 0–2 [19]. The Scheltens’ score was defined as the sum of these ratings (range, 0–30). The assessment of WMLs followed the principle of excluding infarcted or hemorrhagic areas. Additionally, gradient-echo T2*-weighted MRI (GRE) was performed to evaluate the presence of cerebral microbleeds (CMBs). CMBs were defined as homogeneous round lesions with diameters ≤ 10 mm characterized by signal intensity loss on the GRE image. The presence or absence of RVLM vascular compression was evaluated using 3D time-of-flight imaging. The RVLM was located at the root entry zone of cranial nerves IX and X. The upper and lower borders of the root entry zone were determined by the uppermost and lowest fibers of the IX/X nerve bundle entering the medulla; the anterior border of the root entry zone was defined as the transition of the olivary convexity to the concavity of the retro-olivary sulcus; and the posterolateral border was located at the junction of parenchymal brain tissue and individual nerve fiber [16]. Two stroke neurologists (T.N. and F.E.) who were unaware of the clinical details of the patients graded the severity of the PVH (Fazekas rating), DWMH (Fazekas rating) and WMLs (Scheltens’ rating) and identified CMBs and RVLM compression. Joint assessments were conducted by the stroke neurologists (T.N. and F.E.) when necessary to reach a consensus.

### Statistical analysis

Statistical analyses were performed using JMP 17.0 statistical software (SAS Institute, Cary, NC, USA). The data are expressed as the means ± standard deviations (SDs) or medians (25th and 75th percentiles) for the continuous variables and as frequencies and percentages for the discrete variables. The statistical significance of intergroup differences was assessed by the *χ*^*2*^ test, unpaired *t* test, Mann‒Whitney *U* test or Kruskal‒Wallis test, as appropriate. Relationships between the CSA of the VN (right or left) and the other variables and the relationships between WMLs and the other variables were examined by Spearman’s correlation. Indicators of the severity of WMLs were identified using a multiple linear regression model that included the CSA of the right VN and other variables identified by a backward selection procedure using *P* > 0.10 of the likelihood ratio test as the exclusion criterion (Model 1). A similar analysis of the CSA of the left VN was performed (Model 2). *P* < 0.05 indicated statistical significance.

## Results

**Fig. 2:**
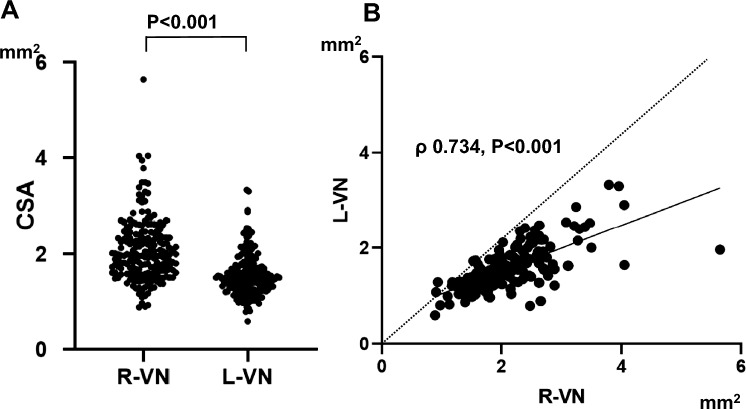
**A** The cross-sectional area (CSA) of the right vagus nerve (VN) was significantly larger than that of the left VN (paired *t* test, *P* < 0.001). **B** The CSA of the right VN was positively correlated with that of the left VN (Spearman’s correlation analysis, *ρ* = 0.734, *P* < 0.001). The presence of nearly all the data points below the identity line suggested that the CSA of the L-VN was smaller than that of the R-VN

A total of 196 patients (128 males and 68 females, 70.2 ± 12.7 years of age) were enrolled in the study. The baseline clinical characteristics of the patients are presented in Table 1. Of these 196 patients, 108 (55.1%) were acute ischemic stroke patients. The CSA of the right VN (2.08 ± 0.65 mm^2^) was significantly greater than the left CSA (1.56 ± 0.44 mm^2^) in these patients (paired *t* test, *t* = 15.80, *P* < 0.001; Fig. 2A). In addition, the CSA of the right VN was positively correlated with the CSA of the left VN (Spearman’s correlation analysis, *ρ* = 0.734, *P* < 0.001; Fig. 2B). The CSAs of both VNs were significantly greater in male patients than in female patients (Table 1). Univariate regression analysis revealed that the CSA of the right VN was significantly correlated with age, male sex, height, weight and BMI (Supplemental Table 1). The associations between the CSA of the left VN and age, sex, and physical findings were similar. Vascular risk factors and atherosclerosis findings in the carotid artery were not associated with the CSA of either VN. Only age was independently associated with the CSAs of both VNs after adjusting for sex and physical findings (height and weight) (Supplemental Table 2). Male sex was associated with the CSAs of both VNs after adjusting for age and BMI (Supplemental Table 2).

**Table 1:** Baseline characteristics

	All *n* = 196	Male *n* = 128	Female *n* = 68	*P*
Age (years)	70.2 ± 12.7	70.5 ± 11.4	69.7 ± 14.8	0.65
Height (cm)	162.0 ± 9.4	166.9 ± 6.5	152.7 ± 6.4	< 0.001
Weight (kg)	60.1 ± 12.9	64.4 ± 10.8	52.2 ± 12.9	< 0.001
Body mass index (kg/m^2^)	22.8 ± 4.0	23.1 ± 3.4	22.3 ± 4.9	0.21
Hypertension	146 (74.5)	96 (75.0)	50 (73.5)	0.86
Diabetes mellitus	60 (30.6)	49 (38.3)	11 (16.2)	0.002
Dyslipidemia	114 (58.2)	72 (56.3)	42 (61.8)	0.54
Atrial fibrillation	28 (14.3)	20 (15.6)	8 (11.8)	0.53
Stroke type				
Acute ischemic stroke	108 (55.1)	71 (55.5)	37 (54.4)	0.95
Acute hemorrhagic stroke	11 (5.6)	7 (5.5)	4 (5.9)	
Chronic stroke (ischemic or hemorrhagic)	23 (11.7)	16 (12.5)	7 (10.3)	
Asymptomatic carotid atherosclerosis	38 (19.4)	23 (18.0)	15 (22.1)	
Others	16 (8.2)	11 (8.6)	5 (7.4)	
Carotid ultrasonography findings				
Right CCA IAD (mm)	7.69 ± 1.00	7.90 ± 0.93	7.32 ± 0.97	< 0.001
Right CCA max-IMT (mm)	1.45 ± 0.97	1.50 ± 0.97	1.35 ± 1.03	0.30
Left CCA IAD (mm)	7.59 ± 0.97	7.79 ± 0.87	7.23 ± 1.04	< 0.001
Left CCA max-IMT (mm)	1.41 ± 0.89	1.47 ± 0.97	1.29 ± 0.74	0.30
CSA of right VN (mm^2^)	2.08 ± 0.65	2.18 ± 0.70	1.89 ± 0.52	0.003
CSA of left VN (mm^2^)	1.56 ± 0.44	1.62 ± 0.46	1.45 ± 0.38	0.011
MRI findings				
PVH (Fazekas rating)	1 (1*–*2)	1 (1*–*2)	1 (1*–*2)	0.82
DWMH (Fazekas rating)	1 (1*–*2)	1 (1*–*2)	1 (0.3*–*2)	0.31
WMLs (Scheltens’ rating)	8 (5*–*2)	8.5 (5*–*12.8)	8 (5*–*12)	0.88
Cerebral microbleeds (*n* = 195)	41 (21.0)	30 (23.6)	11 (16.2)	0.27
Number of cerebral microbleeds (*n* = 195)	0 (0*–*0)	0 (0*–*0)	0 (0*–*0)	0.19
RVLM compression	64 (32.7)	39 (30.5)	25 (36.8)	0.42

The data are presented as the means ± SD for age, height, weight, body mass index, and carotid ultrasonography findings; medians (interquartile ranges) for the Fazekas score, Scheltens’ score and number of cerebral microbleeds; and number (%) for patients

*IAD* interadventitial diameter, *CCA* common carotid artery, *IMT* intima-media thickness, *CSA* cross-sectional area, *MRI* magnetic resonance imaging, *PVH* periventricular hyperintensity, *DWMH* deep white matter hyperintensity, *WMLs* white matter lesions, *RVLM* rostral ventrolateral medulla

**Table 2:** Associations between periventricular hyperintensity (PVH), vascular risk factors, carotid ultrasonography findings, and other magnetic resonance imaging (MRI) findings

	PVH				
	Fazekas 0 (*n* = 33)	Fazekas 1 (*n* = 81)	Fazekas 2 (*n* = 66)	Fazekas 3 (*n* = 16)	*P*
Age (years)	57.2 ± 16.4	70.1 ± 9.4	74.7 ± 9.6	79.6 ± 9.2	< 0.001
Male sex	22 (66.7)	53 (65.4)	39 (59.1)	14 (87.5)	0.20
Height (cm)	164.7 ± 8.2	163.0 ± 9.3	159.2 ± 9.3	162.6 ± 9.9	0.023
Weight (kg)	63.1 ± 13.9	62.4 ± 14.5	56.7 ± 10.1	56.8 ± 8.3	0.019
Body mass index (kg/m^2^)	23.1 ± 4.4	23.3 ± 4.5	22.3 ± 3.0	21.6 ± 3.8	0.25
Hypertension	16 (48.5)	60 (74.1)	57 (86.4)	13 (81.3)	< 0.001
Diabetes mellitus	6 (18.2)	23 (28.4)	26 (39.4)	5 (31.3)	0.17
Dyslipidemia	14 (42.4)	46 (56.8)	43 (65.2)	11 (68.8)	0.14
Atrial fibrillation	1 (3.0)	12 (14.8)	12 (18.2)	3 (18.8)	0.21
Stroke type					
Acute ischemic stroke	17 (51.5)	36 (44.4)	43 (65.2)	12 (75.0)	0.20
Acute hemorrhagic stroke	2 (6.1)	7 (8.6)	2 (3.0)	0 (0.0)	
Chronic stroke (ischemic or hemorrhagic)	2 (6.1)	11 (13.6)	7 (10.6)	3 (18.8)	
Asymptomatic carotid atherosclerosis	9 (27.3)	20 (24.7)	8 (12.1)	1 (6.3)	
Others	3 (9.1)	7 (8.6)	6 (9.1)	0 (0.0)	
Carotid ultrasonography			–	–	
Right CCA IAD (mm)	7.07 ± 1.03	7.69 ± 0.95	7.84 ± 0.91	8.31 ± 1.04	< 0.001
Right CCA max-IMT (mm)	0.97 ± 0.53	1.51 ± 1.14	1.53 ± 0.85	1.72 ± 1.01	0.018
Left CCA IAD (mm)	7.03 ± 1.01	7.62 ± 0.91	7.67 ± 0.96	8.24 ± 0.72	< 0.001
Left CCA max-IMT (mm)	1.08 ± 0.71	1.37 ± 0.93	1.54 ± 0.85	1.73 ± 1.09	0.049
CSA of right VN (mm^2^)	2.24 ± 0.67	2.19 ± 0.70	1.95 ± 0.57	1.76 ± 0.55	0.012
CSA of left VN (mm^2^)	1.68 ± 0.44	1.61 ± 0.44	1.50 ± 0.42	1.31 ± 0.35	0.017
MRI findings					
DWMH (Fazekas rating)	0 (0–1)	1 (1–1)	2 (1–2)	3 (3–3)	< 0.001
WMLs (Scheltens’ rating)	3 (1.5–5)	7 (4–9)	12 (9–15)	19.5 (17–21.8)	< 0.001
Cerebral microbleeds, (*n* = 195)	3 (9.1)	12 (15.0)	17 (25.8)	9 (56.3)	< 0.001
Number of cerebral microbleeds (*n* = 195)	0 (0–0)	0 (0–0)	0 (0–1)	1 (0–3.8)	< 0.001
RVLM compression	7 (21.2)	26 (32.1)	25 (37.9)	6 (37.5)	0.40

The data are presented as the means ± SD for age, height, weight, body mass index, and carotid ultrasonography findings; medians (interquartile ranges) for the Fazekas score, Scheltens’ score and number of cerebral microbleeds; and number (%) for patients

*IAD* interadventitial diameter, *CCA* common carotid artery, *IMT* intima-media thickness, *CSA* cross-sectional area, *MRI* magnetic resonance imaging, *PVH* periventricular hyperintensity, *DWMH* deep white matter hyperintensity, *WMLs* white matter lesions, *RVLM* rostral ventrolateral medulla

### Associations between MRI findings (PVH or DWMH) and carotid ultrasonography findings

Clinical characteristics according to the severity of PVH (Fazekas) are shown in Table 2. Age and hypertension were strongly associated with the severity of PVH. An increased CCA IAD was also associated with PVH, and the CCA max-IMT was slightly associated with PVH. A decreased CSA of the VN was also associated with the severity of PVH. Clinical characteristics according to the severity of DWMH (Fazekas) are shown in Table 3. Like for the PVH, age, hypertension, and CCA IAD were strongly associated with the severity of DWMH. A decreased CSA of the right VN was associated with DWMH (*P* = 0.012), but a decreased CSA of the left VN was not associated with DWMH (*P* = 0.088). Unlike PVH, the CCA max-IMT was not associated with DWMH, while atrial fibrillation and RVLM compression were associated with DWMH (*P* = 0.028 and *P* = 0.032).

**Table 3:** Associations between deep white matter hyperintensity (DWMH), vascular risk factors, carotid ultrasonography findings, and other magnetic resonance imaging (MRI) findings

	DWMH				
	Fazekas 0 (*n* = 37)	Fazekas 1 (*n* = 83)	Fazekas 2 (*n* = 58)	Fazekas 3 (*n* = 18)	*P*
Age (years)	59.0 ± 16.1	69.9 ± 10.4	75.4 ± 7.8	78.5 ± 11.2	< 0.001
Male sex	20 (54.1)	58 (69.9)	36 (62.1)	14 (77.8)	0.23
Height (cm)	162.9 ± 9.4	162.5 ± 8.8	160.7 ± 10.0	161.8 ± 10.0	0.63
Weight (kg)	62.8 ± 14.0	60.9 ± 13.8	58.1 ± 11.4	57.4 ± 9.8	0.25
Body mass index (kg/m^2^)	23.5 ± 4.2	23.0 ± 4.4	22.4 ± 3.4	21.9 ± 3.4	0.42
Hypertension	17 (46.0)	62 (74.7)	51 (87.9)	16 (88.9)	< 0.001
Diabetes mellitus	10 (27.0)	24 (28.9)	21 (36.2)	5 (27.8)	0.74
Dyslipidemia	16 (43.2)	48 (57.8)	38 (65.5)	12 (66.7)	0.16
Atrial fibrillation	3 (8.1)	8 (9.6)	15 (25.9)	2 (11.1)	0.028
Stroke type					0.12
Acute ischemic stroke	15 (40.5)	43 (51.8)	37 (63.8)	13 (72.2)	
Acute hemorrhagic stroke	3 (8.1)	6 (7.2)	2 (3.5)	0 (0.0)	
Chronic stroke (ischemic or hemorrhagic)	4 (10.8)	9 (10.8)	7 (12.1)	3 (16.7)	
Asymptomatic carotid atherosclerosis	10 (27.0)	21 (25.3)	5 (8.6)	2 (11.1)	
Others	5 (13.5)	4 (4.8)	7 (12.1)	0 (0.0)	
Carotid ultrasonography			–	–	
Right CCA IAD (mm)	6.90 ± 0.96	7.78 ± 0.87	7.89 ± 0.95	8.28 ± 1.02	< 0.001
Right CCA max-IMT (mm)	1.31 ± 1.19	1.42 ± 0.96	1.51 ± 0.82	1.66 ± 0.98	0.59
Left CCA IAD (mm)	7.05 ± 1.08	7.66 ± 0.96	7.71 ± 0.88	7.96 ± 0.79	0.002
Left CCA max-IMT (mm)	1.14 ± 0.67	1.46 ± 0.96	1.45 ± 0.84	1.58 ± 1.08	0.22
CSA of right VN (mm^2^)	2.33 ± 0.82	2.11 ± 0.62	1.96 ± 0.57	1.80 ± 0.49	0.012
CSA of left VN (mm^2^)	1.64 ± 0.44	1.59 ± 0.48	1.5 ± 0.39	1.34 ± 0.33	0.088
MRI findings					
PVH (Fazekas rating)	0 (0−1)	1 (1−2)	2 (1−2)	3 (2−3)	< 0.001
WMLs (Scheltens’ rating)	3 (1.5−5)	8 (5−10)	12 (9−15)	19.5 (15.8−22)	< 0.001
Cerebral microbleeds, (*n* = 195)	4 (10.8)	14 (17.1)	14 (24.1)	9 (50.0)	0.006
Number of cerebral microbleeds (*n* = 195)	0 (0−0)	0 (0−0)	0 (0−0.3)	0.5 (0−2.3)	0.004
RVLM compression	7 (18.9)	24 (28.9)	27 (46.7)	6 (33.3)	0.032

The data are presented as the means ± SD for age, height, weight, body mass index, and carotid ultrasonography findings; medians (interquartile ranges) for the Fazekas score, Scheltens’ score and number of cerebral microbleeds; and number (%) for patients

*IAD* interadventitial diameter, *CCA* common carotid artery, *IMT* intima-media thickness, *CSA* cross-sectional area, *MRI* magnetic resonance imaging, *PVH* periventricular hyperintensity, DWMH deep white matter hyperintensity, *WMLs* white matter lesions, *RVLM* rostral ventrolateral medulla

### Associations between WMLs and VN Size

**Fig. 3:**
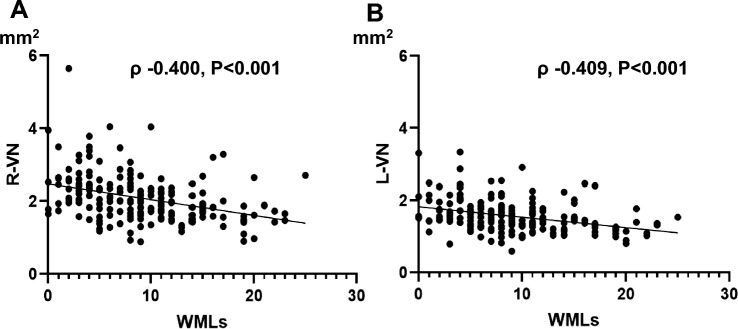
Scatter plots of the vagus nerve (VN) and white matter lesions (WMLs). **A** The CSA of the right VN (R-VN) was negatively correlated with the severity of WMLs (Spearman’s correlation analysis, *ρ* = − 0.400, *P* < 0.001). **B** The CSA of the left VN (L-VN) was negatively correlated with the severity of WMLs (Spearman’s correlation analysis, *ρ* = − 0.409, *P* < 0.001)

Scatter plots of WMLs (Scheltens’ rating score) and the CSA of the VN (right or left) are shown in Fig. 3. The CSAs of both VNs were negatively correlated with the severity of WMLs (right: *ρ* = − 0.400, *P* < 0.001; left: *ρ* − 0.409, *P* < 0.001). Other indicators associated with the severity of WMLs are shown in Table 3. Multiple linear regression analyses revealed that older age (standardized partial regression coefficient [*β*] 0.290; *P* < 0.001), hypertension (β 0.144; *P* = 0.027), increased right CCA IAD (β 0.181; *P* = 0.011), and decreased CSA of the right VN (*β* − 0.226; *P* < 0.001) were independently associated with the severity of WMLs (Model 1). A decreased CSA of the left VN was also associated with the severity of WMLs (*β* = − 0.239; *P* < 0.001, Model 2) (Table 4).

**Table 4:** Indicators of cerebral white matter lesions (WMLs) using univariate or multivariate analysis

	WMLs					
	Spearman’s correlation		Multiple linear regression (Model 1)		Multiple linear regression (Model 2)	
	*ρ*	*P*	*β*	*P*	*β*	*P*
Age (years)	0.525	< 0.001	0.290	< 0.001	0.277	< 0.001
Male sex	0.011	0.88	*− *	*− *	*− *	*− *
Height (cm)	− 0.177	0.013	*− *	*− *	*− *	*− *
Weight (kg)	− 0.213	0.003	− 0.115	0.095	− 0.122	0.075
Body mass index (kg/m^2^)	− 0.149	0.037	*− *	*− *	*− *	*− *
Hypertension	0.294	< 0.001	0.144	0.027	0.150	0.020
Diabetes mellitus	0.131	0.068	*− *	*− *	*− *	*− *
Dyslipidemia	0.105	0.144	*− *	*− *	*− *	*− *
Atrial fibrillation	0.180	0.012	*− *	*− *	*− *	*− *
Carotid ultrasonography			*− *	*− *	*− *	*− *
Right CCA IAD (mm)	0.272	< 0.001	0.181	0.011	0.190	0.007
Right CCA max-IMT (mm)	0.184	0.011	*− *	*− *	*− *	*− *
Left CCA IAD (mm)	0.201	0.005	*− *	*− *	*− *	*− *
Left CCA max-IMT (mm)	0.102	0.16	*− *	*− *	*− *	*− *
CSA of right VN (mm^2^)	− 0.400	< 0.001	− 0.226	< 0.001	*− *	*− *
CSA of left VN (mm^2^)	− 0.409	< 0.001	*− *	*− *	− 0.239	< 0.001
RVLM compression	0.138	0.054	*− *	*− *	*− *	*− *

Model 1. Indicators were identified using multiple linear regression, including the CSA of the right VN and other variables (age, male sex, height, body mass index, hypertension, diabetes mellitus, dyslipidemia, atrial fibrillation, right CCA IAD, right CCA max-IMT, left CCA max-IMT, and RVLM compression), by a backward selection procedure using P > 0.10 of the likelihood ratio test as the exclusion criterion

Model 2. The same analysis as Model 1, incorporating left-VN as an adjusting factor instead of right-VN

*IAD* interadventitial diameter, *CCA* common carotid artery, *IMT* intima-media thickness, *CSA* cross-sectional area, *RVLM* rostral ventrolateral medulla

## Discussion

In this study, age was strongly associated with the CSA of the VN in patients with vascular risk factors. The CSAs of both VNs were independently associated with the severity of WMLs after adjusting for age, sex, vascular risk factors, carotid atherosclerosis and RVLM compression.

Several studies have reported normal values for the CSA of the VN using ultrasonography [3, 20]. In general, the CSA of the right VN is larger than that of the left VN because of the asymmetry in the visceral nerve distribution for cardiac function or the gastric plexus [20, 21]. Our results were consistent with previous findings of differences between the right and left sides. Previous reports have shown that the anatomical level at which measurements are taken affects the CSA of the VN [3]. The CSA of the VN was smaller at the thyroid gland level on both sides than at the common carotid bifurcation level. A recent meta-analysis showed that the mean CSA at the thyroid gland level was 2.39 mm^2^ for the right VN and 1.87 mm^2^ for the left VN [3]. The CSAs of the VNs in our cohort were smaller than the previously reported values. Although there is no definitive consensus on whether factors such as age, sex, height, or weight affect the size of the VN[3, 20, 21], factors such as age and petite body size might influence the size of the VN. In the present study, vascular risk factors, including diabetes mellitus, were not associated with the CSA of the VN. Tawfik et al. reported that patients with diabetes mellitus had a smaller CSA of the VN than did those without diabetes mellitus [6]. On the other hand, associations between decreased VN size and diabetes mellitus were not observed in other previous studies [22, 23]. Although atrial fibrillation was not associated with the CSA of the VN in our cohort, a decreased CSA of the right VN has been associated with the future development of atrial fibrillation in acute ischemic stroke patients [24]. Further evidence is crucial for determining whether these vascular risk factors may be associated with the CSA of the VN.

Our novel findings were that a decreased CSA of the VN was associated with the severity of WMLs. To our knowledge, this study is the first report to demonstrate associations between the CSA of the VN and WMLs and to consider RVLM and atherosclerotic findings in carotid arteries. Older age and hypertension are considered the main risk factors for cerebral WMLs [10, 25]. In this study, older age was strongly associated with decreased VN size. Therefore, older age may be a common etiological factor in the progression of WMLs and atrophy of the VN. Another possible etiology might be autonomic dysfunction, including blood pressure variability. Blood pressure variability was also associated with the severity or progression of WMLs [13, 14]. One limitation of this study was the lack of investigations into blood pressure variability. Diabetes mellitus was also associated with the severity of WMLs [26]. The duration of diabetes may also affect autonomic nerve system dysfunction. In the present study, diabetes mellitus was not associated with the severity of WMLs and the size of VN. Further investigation is needed to determine whether the extent and control status of diabetes duration affect WMLs and VN size.

Recently, imbalances in the gut microbiota have been linked to neurological diseases, such as Parkinson's disease and stroke, indicating a potential brain–gut interaction [27]. In addition, there have recently been several reports on the association between the gut microbiome and cerebral small vessel disease, including WMLs [28, 29]. The connection between the gut microbiota and the brain is thought to involve a complex network, with the VN playing a significant role [30]. It might be worthwhile to explore how the gut microbiota may influence the association between the severity of WMLs and VN size in future investigations.

Interestingly, the factors associated with PVH differed slightly from those associated with DWMH. PVH was associated with large vessel arteriosclerosis, indicated by CCA max-IMT. On the other hand, DWMH was associated with atrial fibrillation and RVLM compression. Several studies have shown that atrial fibrillation is associated with DWMH rather than PVH due to the microembolic pathway [31, 32]. There are limited reports on the correlation between compression of the RVLM and WMLs, but we have previously explored this association in patients with acute ischemic stroke [16]. Among 622 acute ischemic stroke patients, 213 (34.2%) exhibited vascular RVLM compression. Patients with RVLM compression had a greater frequency of severe DWMH with a Fazekas score of 3 than did those without RVLM compression, but this association was not observed for severe PVH with a Fazekas score of 3 [16]. Our current findings demonstrated a similar association between RVLM compression and DWMH to that observed in our previous study. This study might provide valuable insights into the distinct progression mechanisms of PVH and DWMH.

This study has several limitations. First, our study had a small sample size and a cross-sectional design, which limited our ability to make causal inferences. Second, this was a study that retrospectively investigated patients who underwent carotid ultrasound and MRI examinations as part of routine medical care, and it specifically limited the measurement of VN size to three designated technicians. Therefore, there may be selection bias. Third, the majority of our study subjects were acute-phase stroke patients. When considering the association between VN size and blood pressure variability in the future, it may be necessary to also consider healthy individuals. Fourth, all sonographic examinations were performed by three specific, well-trained ultrasound technicians certified by the Japan Academy of Neurosonology. The measurement site for the VN was predetermined through consensus, and interrater reliability was assessed. However, a detailed evaluation of the interrater reliability among the examiners of the patients in this study was not conducted. Fifth, several studies reported that measurements of CSA were performed three times, and mean values were calculated [3, 20]. We measured the CSA of the VN twice and adopted the average, but for a more accurate assessment, it might be better to measure it five times, exclude the highest and lowest values, and then average the remaining three measurements. Sixth, we did not collect other vascular risk factors such as smoking habit, the levels of cholesterol levels and homocysteine levels. These factors were thought to be associated with the severity of WMLs [26]. Finally, most of the subjects in this study were patients with acute stroke or chronic stroke. Therefore, the assessment of WMLs followed the standard practice of excluding infarcted or hemorrhagic areas. However, there were cases where this evaluation was challenging. It will be worth investigating the causal relationship between WMLs and VN size among patients without a history of stroke.

In conclusion, VN size determined via ultrasonography was associated with the severity of WMLs. While these findings do not establish a causal relationship, they suggest that autonomic nervous system dysfunction is involved in the progression of WMLs.

### Supplementary Information

Below is the link to the electronic supplementary material.

Supplementary file1 (DOC 176 KB)

## Data Availability

The data that supported the findings of this study are available from the corresponding author upon reasonable request.
