# Supplementary material for: Vagus nerve size determined via ultrasonography is associated with white matter lesions in patients with vascular risk factors
Source: J Ultrasound. 2024 Jul 29;27(3):723–32. doi: 10.1007/s40477-024-00936-2 (PMC11333691; doi:10.1007/s40477-024-00936-2)
Supplement: Supplementary file 1 — Supplementary file1 (DOC 176 KB) [file 40477_2024_936_MOESM1_ESM.doc]

Supplemental Table 1. Associations between vascular risk factors and vagus nerve size

|  | Right vagus nerve | | Left vagus nerve | | |
| --- | --- | --- | --- | --- | --- |
|  | ρ | P | ρ | P | |
| Age (years) | -0.337 | <0.001 | -0.364 | | <0.001 |
| Male sex | 0.223 | 0.002 | 0.195 | | 0.006 |
| Height (cm) | 0.315 | <0.001 | 0.263 | | <0.001 |
| Weight (kg) | 0.294 | <0.001 | 0.253 | | <0.001 |
| Body mass index (kg/m^2^) | 0.170 | 0.018 | 0.140 | | 0.051 |
| Hypertension | -0.102 | 0.15 | -0.087 | | 0.23 |
| Diabetes mellitus | -0.078 | 0.28 | 0.022 | | 0.76 |
| Dyslipidemia | -0.102 | 0.18 | -0.096 | | 0.18 |
| Atrial fibrillation | -0.040 | 0.58 | -0.041 | | 0.57 |
| **Carotid ultrasonography** |  |  |  | |  |
| Right CCA IAD (mm) | 0.014 | 0.85 | 0.022 | | 0.77 |
| Right CCA max-IMT (mm) | -0.058 | 0.43 | -0.085 | | 0.24 |
| Left CCA IAD (mm) | 0.067 | 0.35 | 0.078 | | 0.28 |
| Left CCA max-IMT (mm) | -0.042 | 0.57 | 0.039 | | 0.59 |

IAD, interadventitial diameter; CCA, common carotid artery; IMT, intima-media thickness

Supplemental Table 2. Associations between age, sex, and physical findings and vagus nerve size according to multiple linear regression analysis

|  | Right vagus nerve | | Left vagus nerve | |
| --- | --- | --- | --- | --- |
|  | β | P | β | P |
| Age (years) | -0.333 | <0.001 | -0.337 | <0.001 |
| Male sex | 0.144 | 0.15 | 0.170 | 0.094 |
| Height (cm) | 0.114 | 0.30 | 0.018 | 0.87 |
| Weight (kg) | -0.001 | 0.94 | 0.025 | 0.78 |

|  | Right vagus nerve | | Left vagus nerve | |
| --- | --- | --- | --- | --- |
|  | β | P | β | P |
| Age (years) | -0.360 | <0.001 | -0.347 | <0.001 |
| Male sex | 0.226 | <0.001 | 0.194 | 0.004 |
| BMI (kg/m^2^) | -0.014 | 0.83 | 0.010 | 0.89 |

β is the standardized partial regression coefficient.

IAD, interadventitial diameter; CCA, common carotid artery; IMT, intima-media thickness
